# Melatonin inhibits muscular-mucosal stretch-sensitive bladder afferents via the MT2 receptors

**DOI:** 10.1038/s41598-022-22705-z

**Published:** 2022-10-21

**Authors:** Stewart Ramsay, Vladimir Zagorodnyuk

**Affiliations:** Discipline of Human Physiology, Flinders Health and Medical Research Institute, College of Medicine and Public Health, Flinders University, GPO Box 2100, Adelaide, SA Australia

**Keywords:** Neuroscience, Urology

## Abstract

Melatonin is a circadian rhythm regulator capable of controlling a variety of physiological processes in the body. It predominantly acts via the melatonin 1 (MT1) and MT2 receptors expressed in the CNS neurons and peripheral organs and tissues. Melatonin can modulate urinary bladder function, however, to date it is not known if melatonin can regulate activity of sensory neurons innervating the bladder. Bladder afferents play an important role in urine storage and voiding. Therefore, this study aims to determine if melatonin can regulate mechanosensitivity of 2 major classes of sensory neurons in the guinea pig bladder: stretch-insensitive mucosal and low threshold stretch-sensitive muscular-mucosal afferents. The effects of melatonin on the mechanosensitivity of mucosal and muscular-mucosal afferents were measured ex vivo using single unit extracellular recording. Melatonin did not affect the responses of mucosal afferents to stroking of their receptive fields but did concentration-dependently, significantly inhibit 69% of muscular-mucosal afferents responses to stroking and bladder stretch. This inhibitory effect was not affected by the MT1 receptor antagonist, S26131 but was blocked by the selective MT2 receptor antagonists, K-185 and 4-P-PDOT. Forskolin significantly potentiated the responses of muscular-mucosal afferents to stroking and stretch, which were prevented by melatonin. These findings demonstrate a direct inhibitory effect of melatonin on the mechanosensitivity of low threshold stretch-sensitive muscular-mucosal bladder afferents acting via MT2 receptors, which is independent from its action on detrusor muscle. This may have important clinical implications for the treatment of many common bladder disorders including nocturia.

## Introduction

The circadian system powerfully modulates 24 h rhythms of all body physiological processes, optimising efficiency and coordinating bodily functions including urination^1^. Circadian rhythms are orchestrated by a transcriptional-translational feedback loop for a set of oscillating circadian ‘clock’ genes within the “master clock” located in a region of the anterior hypothalamus, the suprachiasmatic nucleus (SCN)^2,3^. The central master clock can influence the circadian systems found in most peripheral tissues and organs (termed “peripheral clocks”) such as gut, liver, adipose tissue, immune cells, bladder, and others^1,2,4–7^. The hormone melatonin (*N*-acetyl-5-methoxytryptamine) is produced and released primarily during darkness by pinealocytes of the pineal gland and is involved in the synchronisation of the peripheral clocks by the central clock and serves as feedback mechanisms for SCN neurons^8,9^. Melatonin is a powerful regulator of circadian rhythms with concentrations in the blood and urine peaking during the night, thus stabilising sleep–wake cycle. It can regulate a wide variety of physiological processes in mammals^10–12^. However, melatonin is not exclusively a pineal hormone. Many other organs and tissues such as the retina, liver, gut, kidney, and others can synthetise melatonin locally, which then can act as paracrine factor^13–17^.

Melatonin binds the high-affinity G-protein coupled receptors, melatonin 1 (MT1) or MT2 receptors which are expressed on plasma membranes in the central nervous system and in peripheral tissues such as the heart, lung, gut, kidneys, bladder, and immune cells^8,10,11^. Since melatonin is membrane permeable, some of the action of melatonin is due to its effects on nuclear receptors of the ROR family or directly on cytosolic enzymes and Ca^2+^ binding proteins^10,11,18^.

There are indications to suggest that melatonin can ameliorate age-related changes in bladder function^19,20^. Intracerebral administration of melatonin increased bladder capacity in adult rats; this effect was mediated via brain GABAergic neurons^21^. Ex vivo studies demonstrated that melatonin suppressed detrusor contractions induced by electrical stimulation, high K^+^, and L-type Ca^2+^ channel agonist. This inhibition of contractions, however, was not blocked by MT1/MT2 receptor antagonism^22^.

To date, there is no data on the effects of melatonin on bladder sensory neurons. Sensory neurons innervating the bladder play a key role in urine storage and voiding^23^. It is possible that they may involve in circadian rhythm modulation of bladder function. Currently, there are 5 different types of bladder afferents described: mucosal, muscular, muscular-mucosal, vascular (serosal), and silent. Stretch-sensitive muscular and muscular-mucosal afferent types can be divided further into low and high thresholds functional classes of bladder afferents^23–25^.

Our recent study indicated a potential circadian rhythm of at least 3 classes of bladder afferents, mucosal, and low and high threshold muscular-mucosal afferents. All these afferents demonstrated an increased sensitivity to mechanical stimuli (stroking or stretch) during the day and decreased sensitivity during the night in guinea pigs^26^. Given the role of melatonin in the regulation of circadian rhythms, the aim of this study was to investigate whether melatonin can modulate mechanosensitivity of mucosal and low threshold muscular-mucosal afferents in the guinea pig bladder.

## Results

### Melatonin does not affect bladder mucosal afferent sensitivity

**Figure 1:**
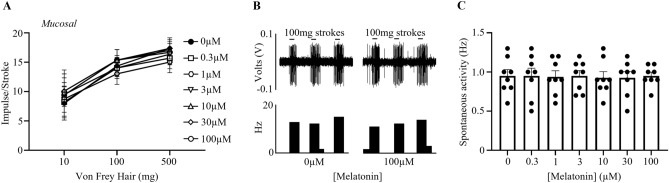
Melatonin does not affect the mechanosensitivity of mucosal bladder afferents. (**A**) The responses of bladder mucosal afferents to von Frey hair stroking (10, 100, and 500 mg) in the absence and presence of melatonin (0.3–100 μM). (**B**) A raw trace example of bladder mucosal afferent responses to stroking (100 mg) in the absence or presence of melatonin (100 μM). (**C**) The spontaneous activity of mucosal afferents in the absence and presence of melatonin (0.3–100 µM). Data is presented as the mean ± the SEM, N = 8, n = 8.

The effect of melatonin on mucosal bladder afferent mechanosensitivity is illustrated in Fig. 1. A total of 8 mucosal afferents were tested for their responsiveness to melatonin (0.3–100 µM). At all concentrations studied (0.3–100 μM), melatonin did not affect the mechanosensitivity of bladder mucosal afferents to stroking (Fig. 1A,B; 100 mg: control 14.3 ± 0.6 impulses/stroke, 100 µM 15.3 ± 0.7 impulses/stroke, NS, N = 8, n = 8).

The spontaneous activity of mucosal bladder afferents was also not affected by melatonin at all concentrations (0.3–100 µM; Fig. 1C; control 0.9 ± 0.08 Hz, 100 µM 1.0 ± 0.04 Hz, NS, N = 8, n = 8).

### Melatonin concentration-dependently inhibits mechanosensitivity of low threshold muscular-mucosal afferents

**Figure 2:**
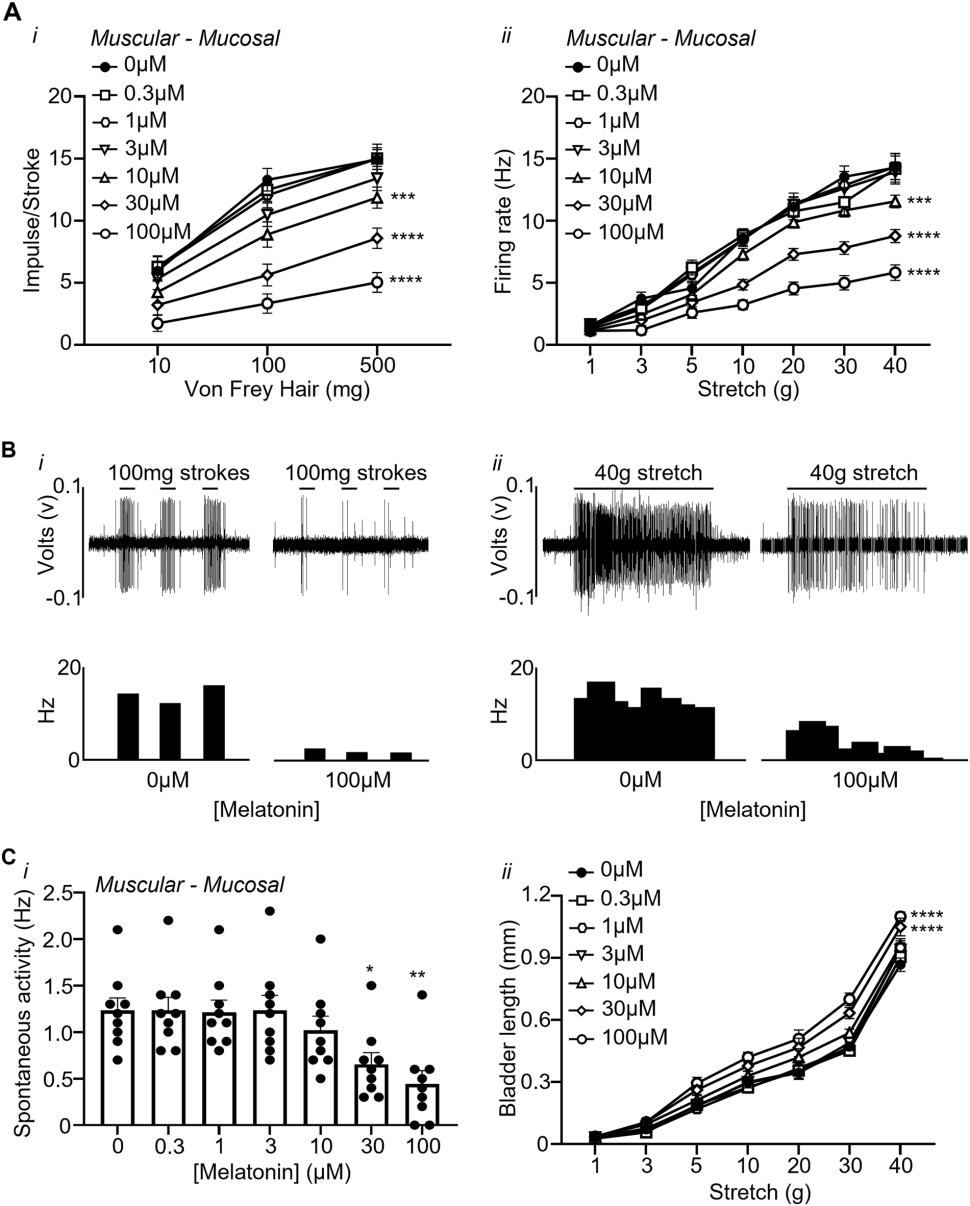
Melatonin inhibits the mechanosensitivity of muscular-mucosal bladder afferents. (**A**) The responses of low threshold muscular-mucosal afferents to (**i**) stroking (10, 100 and 500 mg) and (**ii**) stretch (1–40 g) in the absence or presence of melatonin (0.3–100 μM). (**B**) Raw trace examples of the response of melatonin to (**i**) stroking (100 mg) and (**ii**) stretch (40 g) in the absence and presence of melatonin (100 µM). (**C**) The effect of melatonin on the (**i**) spontaneous activity of muscular-mucosal bladder afferents and (**ii**) bladder length in the absence and presence of melatonin (0.3–100 µM). Data is presented as the mean ± the SEM, N = 8, n = 9. *P < 0.05, **P < 0.01, ***P < 0.001, ****P < 0.0001 vs 0 µM melatonin.

The effect of melatonin on low threshold muscular-mucosal bladder afferent mechanosensitivity and spontaneous activity is illustrated in Fig. 2. A total of 13 low-threshold muscular-mucosal afferents (N = 8) were tested for their responsiveness to melatonin. Of these 13 low threshold muscular-mucosal afferents, 9 responded to melatonin (69%) and 4 did not (31%).

Melatonin (0.3–100 µM) had no significance effects on the response of non-responding muscular-mucosal afferents to stroking (10, 100, and 500 mg) and stretch (1, 3, 5, 10, 20, 30, and 40 g) (stroking 100 µM: 10 mg − 18.7 ± 9.1%, NS, 100 mg − 10.2 ± 5.1%, NS, 500 mg − 12.8 ± 1.1%, NS; stretch 100 µM: 1 g − 1.8 ± 1.8%, NS, 3 g − 22.9 ± 10.5%, NS, 5 g − 14.5 ± 6.6%, NS, 10 g − 9.2 ± 7.2%, NS, 20 g − 9.5 ± 7.2%, NS, 30 g − 1.9 ± 8.1%, NS, 40 g − 22.3 ± 4.4%, NS, N = 3, n = 4).

At concentrations 10–100 μM melatonin concentration-dependently inhibited the mechanosensitivity of responding low threshold muscular-mucosal afferents to stroking (10–500 mg) with maximal effect at 100 µM [Fig. 2Ai,Bi; 100 µM: 10 mg − 77.6 ± 6.9%, P < 0.05, 100 mg − 76.8 ± 4.2%, P < 0.0001, 500 mg − 67.7 ± 3.5%, P < 0.0001, N = 8, n = 9; von Frey hair effect (F(2,168) = 120.9, P < 0.0001), melatonin effect (F(6,168) = 36.4 P < 0.0001), and an interaction (F(12,168) = 1.9 P < 0.05)]. Similarly, melatonin also concentration-dependently (10–100 μM) reduced the mechanosensitivity of low threshold muscular-mucosal afferents to stretch (10–40 g) with maximal effect at 100 µM [Fig. 2Aii,Bii; 100 µM: 10 g − 63.2 ± 2.6%, P < 0.0001, 20 g − 59.8 ± 2.2%, P < 0.0001, 30 g − 63.8 ± 2.0%, P < 0.0001, 40 g − 59.9 ± 1.8%, P < 0.0001, N = 8, n = 9; load effect (F(6,392) = 294.7, P < 0.0001), melatonin effect (F(6,392) = 65.43, P < 0.0001), and an interaction, (F(36,392) = 4.05, P < 0.0001)].

The spontaneous activity of muscular-mucosal afferents was significantly reduced at 30 μM and 100 μM of melatonin [Fig. 2Ci; 30 µM − 49.9 ± 3.7%, P < 0.05, 100 µM − 69.6 ± 7.2%, P < 0.01, N = 8, n = 9; melatonin effect (F(6,56) = 5.302, P < 0.001)]. Melatonin (30, 100 μM) also significantly increased bladder compliance at 20 g, 30 g, and 40 g, measured as a change in length during imposed loads, [Fig. 2Cii; 100 µM: 20 g + 47.4 ± 0.4%, P < 0.001, 30 g + 48.3 ± 0.7%, P < 0.0001, 40 g + 46.7 ± 0.2%, P < 0.0001, N = 8; load effect [(F(6,196) = 1079, P < 0.0001), melatonin effect (F(6,96) = 27.91 P < 0.0001), and an interaction (F(36,196) = 1.84, P < 0.05)].

### The inhibitory effect of melatonin on bladder afferents was abolished by MT2, but not MT1, receptor antagonists

**Figure 3:**
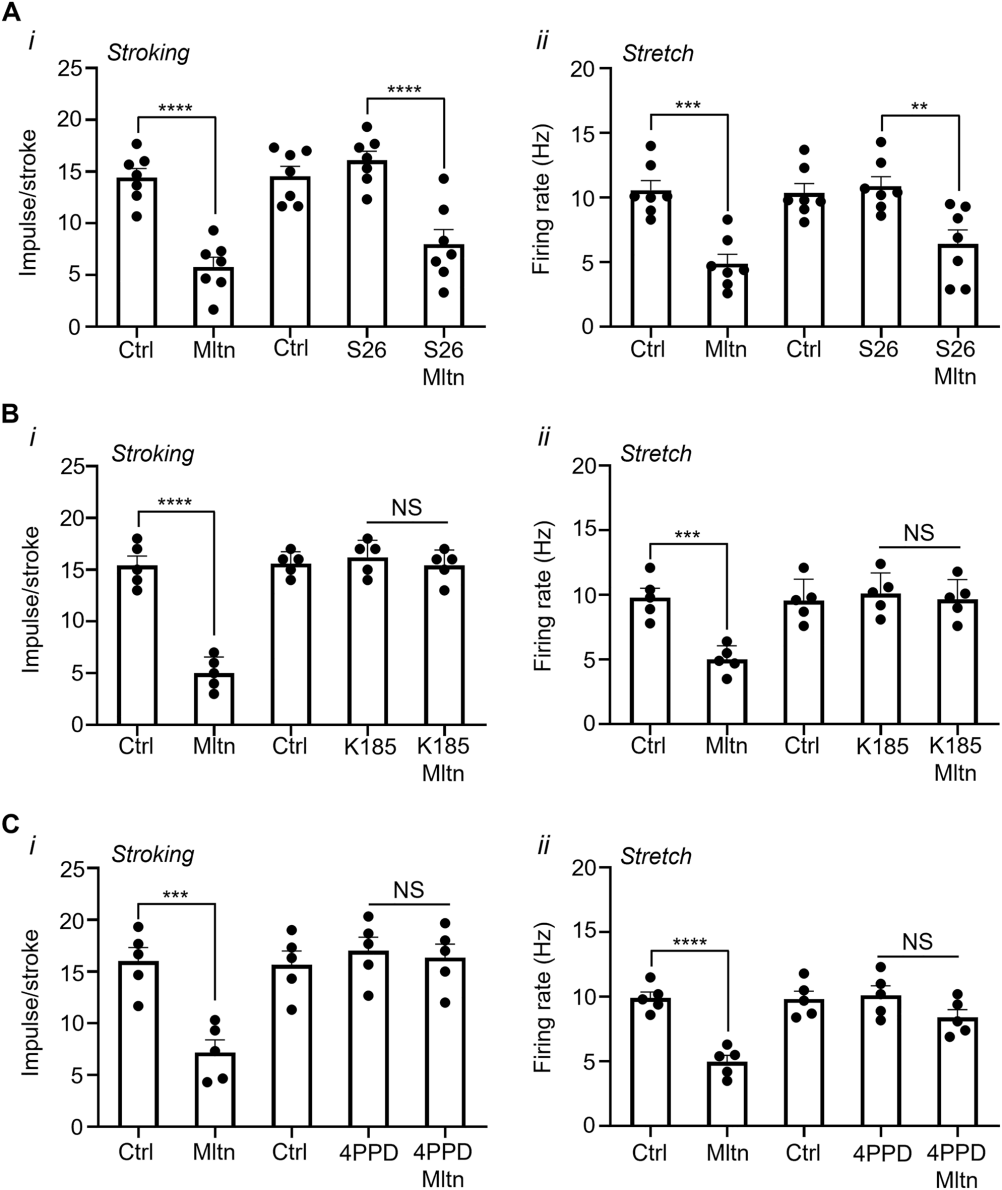
The inhibitory effect of melatonin on muscular-mucosal afferents was abolished by MT2, but not MT1 receptor antagonists. (**A**) The responses of responding bladder muscular-mucosal afferents to (**i**) stroking (100 mg) and (**ii**) stretch (10 g) in the absence and presence of melatonin (Mltn) alone (30 µM) or in combination with the MT1 receptor antagonist, SR26131 (S26; 10 µM). Data is presented as the mean ± the SEM, N = 7, n = 7. **P < 0.01, ***P < 0.001, ****P < 0.0001. (**B**) The effect of melatonin (Mltn; 30 µM) on the responses of muscular-mucosal afferents to (**i**) stroking (100 mg) and (**ii**) stretch (10 g) in the absence and presence of the MT2 receptor antagonist, K-185 (10 µM). (**C**) The effect of melatonin (30 µM) on responses of muscular-mucosal afferents to (**i**) stroking (100 mg) and (**ii**) stretch (10 g) in the absence and presence of the MT2 receptor antagonist, 4-P-PDOT (4PPD; 10 µM). Data is presented as the mean ± the SEM, N = 5, n = 5 per antagonist. ***P < 0.001, ****P < 0.0001 vs control.

The effect the melatonin alone or in combination with the MT1 receptor antagonist S26131, or the MT2 receptor antagonists K185 or 4-P-PDOT, on responding muscular-mucosal bladder afferents is illustrated in Fig. 3. Melatonin (30 µM) significantly inhibited the mechanosensitivity of responding muscular-mucosal bladder afferents to stroking and stretch (Fig. 3Ai,Aiii; stroking − 61.35 ± 4.5%, P < 0.0001, stretch − 55.2 ± 3.6%, P < 0.001, N = 7, n = 7).

MT1 antagonist, SR26131 (10 μM) alone did not significantly affect the mechanosensitivity of low threshold muscular-mucosal bladder afferents to stroking (100 mg) or stretch (10 g) (Fig. 3A). In the presence of the MT1 antagonist SR26131 (10 μM), the inhibitory effect of melatonin remained (stroking − 50.6 ± 8.4%, P < 0.0001, stretch − 41.4 ± 9.1%, P < 0.005, N = 7, n = 7) and was not significantly different from the inhibitory effect of melatonin without the MT1 antagonist (Fig. 3A).

**Figure 4:**
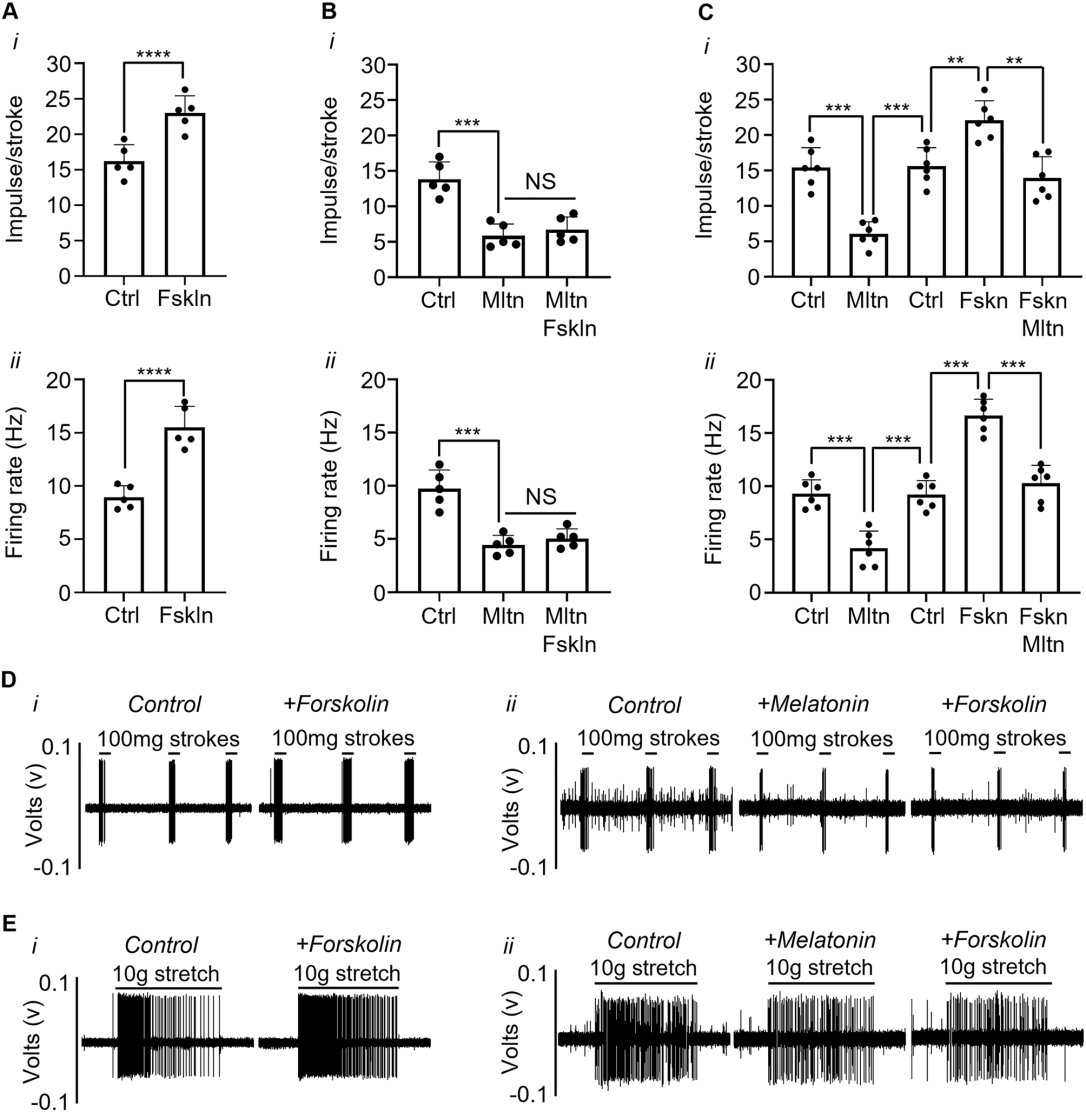
Melatonin prevents forskolin-induced increase in mechanosensitivity of muscular-mucosal bladder afferents. (**A**) The effect of forskolin (Fskln; 10 µM) on the responses of muscular-mucosal afferents to stroking (100 mg) (**i**) and (**ii**) stretch (10 g) in the presence of forskolin (10 µM). (**B**) The responses of muscular-mucosal afferents to (**i**) stroking (100 mg) or (**ii**) stretch (10 g) to melatonin (30 µM) alone or in the addition of forskolin (10 µM). (**C**) The response of muscular-mucosal afferents to (**i**) stroking (100 mg) or (**ii**) stretch (10 g) in the presence of forskolin (10 µM) alone or in the addition of melatonin (30 µM). Data is presented as the mean ± the SEM, N = 5–6, n = 5–6 per group. **P < 0.01, ***P < 0.001, ****P < 0.0001 vs control. (**D**) Raw traces of the response of muscular-mucosal afferents to stroking (100 mg) in (**i**) the presence of forskolin (10 µM) alone or (**ii**) in the presence of both melatonin (30 µM) and forskolin (10 µM). (**E**) Raw traces of the response of muscular-mucosal afferents to stretch (10 g) in (**i**) the presence of forskolin (10 µM) alone or (**ii**) in the presence of both melatonin (30 µM) and forskolin (10 µM).

The MT2 receptor antagonist, K-185 (10 μM) alone did not significantly affect the mechanosensitivity of low threshold muscular mucosal afferents to stroking (100 mg) or stretch (10 g) (Fig. 3Bi,Bii). Melatonin significantly inhibited the responses of muscular-mucosal afferents to stroking and stretch (Fig. 4Ai,Aii; stroking − 67.0 ± 5.5%, P < 0.0001, stretch − 48.8 ± 3.7% P < 0.001, N = 5, n = 5). However, the inhibitory effect of melatonin (30 µM) on stroking and stretch-induced firing was abolished in the presence of the MT2 inhibitor K-185 (Fig. 3Bi,Bii; stroking − 4.8 ± 1.2%, NS, stretch − 4.3 ± 0.6%, NS, N = 5, N = 5).

A different MT2 receptor antagonist, 4-P-PDOT (10 µM), alone, did not significantly affect the mechanosensitivity of responding low threshold muscular-mucosal afferents to stroking (100 mg) or stretch (10 g) (Fig. 3Ci,Cii). The inhibitory effect of melatonin on responses of muscular-mucosal bladder afferents to stroking (100 mg) and stretch (10 g) was present (stroking − 54.8 ± 6.4%, P = 0.0009, stretch − 49.8 ± 4.2%, P < 0.0001, N = 5, n = 5), and was abolished in the presence 4-P-PDOT (Fig. 3Ci,Cii; stroking − 4.0 ± 0.4%, NS, stretch − 10.5 ± 7.1%, NS, N = 5, n = 5).

### Melatonin abolished the forskolin-induced increase in mechanosensitivity of bladder afferents

The effects of melatonin on low threshold muscular-mucosal bladder afferents alone or in combination with forskolin are illustrated in Fig. 4.

The adenylyl cyclase agonist forskolin (10 µM) alone significantly increased the responses of muscular-mucosal afferents to stroking (100 mg) and stretch (10 g) (Fig. 4Ai,Aii; stroking + 42.6 ± 3.9%, P < 0.0001, stretch + 73.7 ± 2.2%, P < 0.0001, N = 5, n = 5). Forskolin alone also significantly increased bladder compliance measured as a change in length during imposed load (10 g) (+ 29.9 ± 4.2%, P < 0.01, N = 5, n = 5). Melatonin alone (30 µM) significantly inhibited the mechanosensitivity of low threshold muscular-mucosal afferents in response to stroking (100 mg) and stretch (10 g) (Fig. 4Bi,Bii; stroking − 58.1 ± 2.8% P < 0.001, stretch − 54.2 ± 3.4%, P < 0.01, N = 5, n = 5). There was no potentiation of the mechanosensitivity of muscular-mucosal afferents to stroking and stretch by forskolin (10 µM) in the presence of melatonin (30 µM). Responses to stroking and stretch were not significantly different between melatonin alone and in the presence of melatonin and forskolin (Fig. 4Bi,Bii; in the presence of melatonin plus forskolin stroking − 51.7 ± 3.2%, P < 0.001, stretch − 47.5 ± 3.3%, P < 0.01, N = 5, n = 5, NS from melatonin alone).

The spontaneous activity of low threshold muscular-mucosal afferents was significantly increased in the presence of forskolin (10 µM) alone (+ 59.3 ± 15.9%, P < 0.001, N = 5, n = 5), which was prevented by melatonin (30 µM). Spontaneous firing of low threshold muscular-mucosal afferents was not significantly different between melatonin alone and in the presence of melatonin and forskolin (in the presence of melatonin − 50 ± 1.7%, P < 0.001, N = 5, n = 5 and in the presence of melatonin plus forskolin − 44.1 ± 3.9%, P < 0.001, N = 5, n = 5, NS).

In a separate set of experiments forskolin was added to the organ bath first followed by melatonin. Melatonin alone (30 µM) significantly inhibited the response of low-threshold muscular mucosal afferents to stroking and stretch compared to controls (Fig. 4Ci,Cii; stroking − 60.6 ± 4.1%, P < 0.001; stretch − 55.7 ± 5.9%, P < 0.001, N = 6, n = 6). Forskolin alone significantly potentiated the response of low-threshold muscular mucosal afferents to stroking and stretch compared to control (Fig. 4Ci,Cii; stroking + 43.1 ± 8.3%, P < 0.01; stretch + 82.8 ± 7.7%, P < 0.001, N = 6, n = 6). In the presence of forskolin, melatonin significantly reduced the response of low-threshold muscular mucosal afferents to stroking and stretch compared to forskolin alone (Fig. 4Ci,Cii; stroking − 36.7 ± 5.2%, P < 0.01; stretch − 38.3 ± 3.3%, P < 0.001, N = 6, n = 6).

In a final set of experiments, forskolin and the MT2 receptor antagonist 4-P-PDOT were added in combination first followed by melatonin. Combination of forskolin (10 µM) and 4-P-PDOT (10 µM) completely abolished the inhibitory effect of melatonin (30 µM; melatonin in presence of forskolin and 4-P-PDOT: stroking 100 mg − 7.9 ± 2.7%, NS, stretch 10 g − 3.4 ± 1.1%, NS, N = 3, n = 3).

## Discussion

Both a hormone and tissue factor, melatonin is a pleotropic substance, capable of controlling a variety of physiological processes in the body^11^. However, no effect melatonin on visceral afferents has been reported to date. The present finding is the first report demonstrating the ability of melatonin to directly regulate bladder afferent mechanosensitivity.

As seen in most mammals, including humans, urinary voiding exhibits a circadian rhythm with increased occurrence during active phases and decreased occurrence during inactive phases^1,6,27,28^. Many parameters of micturition related to functional bladder capacity in both humans and laboratory animals have significant circadian rhythm variation such as urine volume, voiding frequency and voiding volume per void^1,29–32^. Sensory neurons innervating the bladder play a key role in urine storage, micturition, and development of lower urinary symptoms such as pain, urgency, frequency, and nocturia in bladder diseases^23^. We have recently revealed that some classes of bladder afferents, stretch-insensitive mucosal, and low and high threshold stretch-sensitive muscular-mucosal afferents exhibit a potential circadian rhythm modulation: stroking- and stretch-induced firing were increased during the day and decreased during the night in guinea pigs^26^. The mechanism of these powerful changes in mechanosensitivity of bladder afferents is still not clear. One of the potential factors that could be involved is the hormone and tissue paracrine mediator, melatonin, whose plasma and urine levels peak during the night and fall during daytime^12^.

In vivo, centrally administrated melatonin (0.4–40 pM) increases rat bladder capacity. This inhibitory effect on micturition pathways in the brain likely involved GABAergic neurons since GABA_A_ antagonist, bicuculline inhibited the melatonin-induced increase in bladder capacity^21^. It is still unclear whether this melatonin effect is due to activation of MT1/MT2 receptors on GABAergic interneurons or via intracellular nuclear melatonin receptors and/or enzymes. There is little information regarding the direct effects of melatonin on the bladder itself or on the autonomic nerves that control bladder function. In ex vivo, melatonin (0.01–1 µM), in a concentration-dependent manner, inhibited contractions of isolated rat detrusor muscle strips evoked by electrical field stimulation, and high K^+^ or L-type Ca^2+^ channel opener, Bay K 8644^22^. Interestingly, this effect was not affected by non-selective MT1/MT2 melatonin receptor antagonist luzindole, suggesting possible intracellular mechanism of action. In the guinea pig bladder, melatonin also inhibits high K^+^- and acetylcholine-induced contractions of the detrusor strips with IC_50_ close to 100 µM^33^, indicating significant species differences. Present data demonstrated that melatonin affects detrusor muscle compliance in the guinea pig bladder at 30 and 100 µM, but not at 10 µM. However, 10 µM melatonin significantly inhibited mechanosensitivity of muscular-mucosal afferents: both stretch- and stroking-induced responses were significantly reduced. Low threshold muscular-mucosal afferents are stretch-sensitive in-series tension receptors, so they increase firing when length of the bladder tissue is increased^34,35^. It has been demonstrated that intravesical tension developed during slow bladder filling in mice is generated predominantly by passive resistance of the urothelial layer and connective tissue of the lamina propria rather than by active contractions of the smooth muscle cells of the detrusor since pressure–volume relation between intact bladder and bladder wall without detrusor are quite similar^36^. We have previously established that the major stimulus for inducing firing of low threshold bladder afferents in guinea pigs was passive increase in tension (that was confirmed in 0Ca^2+^ 1 mM EDTA Krebs solution), while active contractions added relatively little to overall distension-induced firing^34,35,37^. If melatonin did not have direct inhibitory effects on bladder afferents via MT2 receptors, the melatonin-induced increase in bladder compliance (increased lengthening of the bladder preparation by constant load) at high doses will produce an increase (not a decrease) of stretch-induced firing of bladder afferents. Thus, the inhibitory effect of melatonin on stretch-induced firing (accompanied by an increase in bladder compliance) and its inhibitory effect on stroking-induced firing clearly indicates that melatonin has a direct effect on the mechanosensitivity of this class of bladder afferents inhibiting sensory signalling from the bladder, independently from its effect on detrusor muscle. This new mechanism of melatonin action is important when considering the use of melatonin as potential pharmacological agent to treat detrusor instability but also excessive urgency which could be independent from involuntary bladder contractions.

The inhibitory action of melatonin on muscular-mucosal afferents is through the MT2 G-protein coupled receptors since MT2 antagonists, 4-P-PDOT and K-185, but not MT1 antagonist S26131^38^, were able to abolish it. The data agrees with recent findings on the mouse dorsal root ganglia (DRG), demonstrating MT2 receptor expression on DRG neurons, while MT1 receptors were present on glial cells^39^. Activation of MT1 and MT2 receptors involves multiple signal transduction pathways and vary significantly between different tissues and cell types. The cAMP second messenger system is the most common intracellular pathway involved in melatonin action since activation of MT1 and MT2 receptors inhibits forskolin-stimulated cAMP production in many tissues and cells^8,40,41^. It is known that forskolin relaxes detrusor smooth muscle^42^. This was confirmed in the present study since forskolin increased bladder compliance by 30%. At the same time, forskolin evoked an increase in spontaneous firing of muscular-mucosal afferents and in their responses to stretch (by 73%) and mucosal stroking. This clearly shows that increase in excitability of muscular-mucosal afferents by forskolin is independent of its relaxing effect on the detrusor. The present data indicates that melatonin prevents forskolin-induced increase in spontaneous activity and mechanosensitivity of muscular-mucosal bladder afferents. This suggests that one of the possible mechanisms of the inhibitory action of melatonin on bladder afferents may involve a decrease in cAMP levels. This needs further investigation. Interestingly, low threshold mucosal mechanoreceptors, which are not activated by bladder stretch but are sensitive to mucosal stroking of their receptive fields with von Frey hairs^34,35^, were not affected by melatonin up to 100 µM. These mucosal afferents are likely peptidergic nociceptors since they contain both SP and CGRP and are capsaicin-sensitive in the guinea pig bladder^23,25^. In contrast, low threshold muscular-mucosal afferents are not capsaicin-sensitive and most likely involved in physiological reflexes including storage and micturition.

Notwithstanding that the maximum concentration of melatonin in the blood serum at night is around 1 nM in humans^43^, many investigators consider a concentration range of 0.1–1 µM as physiological since concentration of melatonin in many biological fluids and organs up to 2–3 orders of magnitude higher than that in the serum^13,44–47^. This is because many organs and tissues such as the retina, liver, gut, kidney, skin, testes, immune cells can synthetise melatonin locally^13–17^, which then can act intracellularly or as paracrine tissue mediator. The paracrine action of peripherally produced melatonin most likely includes physiological regulation of cell homeostasis, due to its profound general antioxidative actions, but also other tissue specific effects^10,13,48,49^. To date, there is no data available regarding whether melatonin can be locally produced by urothelial cells, interstitial cells or other cell types within the bladder wall. One potential source of locally produced melatonin in the bladder is immune cells. In the bladder, there is dense network of resident mast cells in the lamina propria and detrusor muscle^50^. It is well established that mast cells and other immune cells express MT1 and MT2 receptors and are capable of synthesising melatonin^46^. Therefore, a possible role of locally produced melatonin within bladder itself, which then can cause inhibition of the mechanosensitivity of bladder afferents, cannot be ruled out at present. Importantly, the effective doses (2–10 mg) of exogenously taken melatonin against insomnia in adults and nocturia symptoms in the elderly^20,51–54^ are in a similar range in which inhibitory effects on bladder afferents detected in guinea pig bladder.

**Figure 5:**
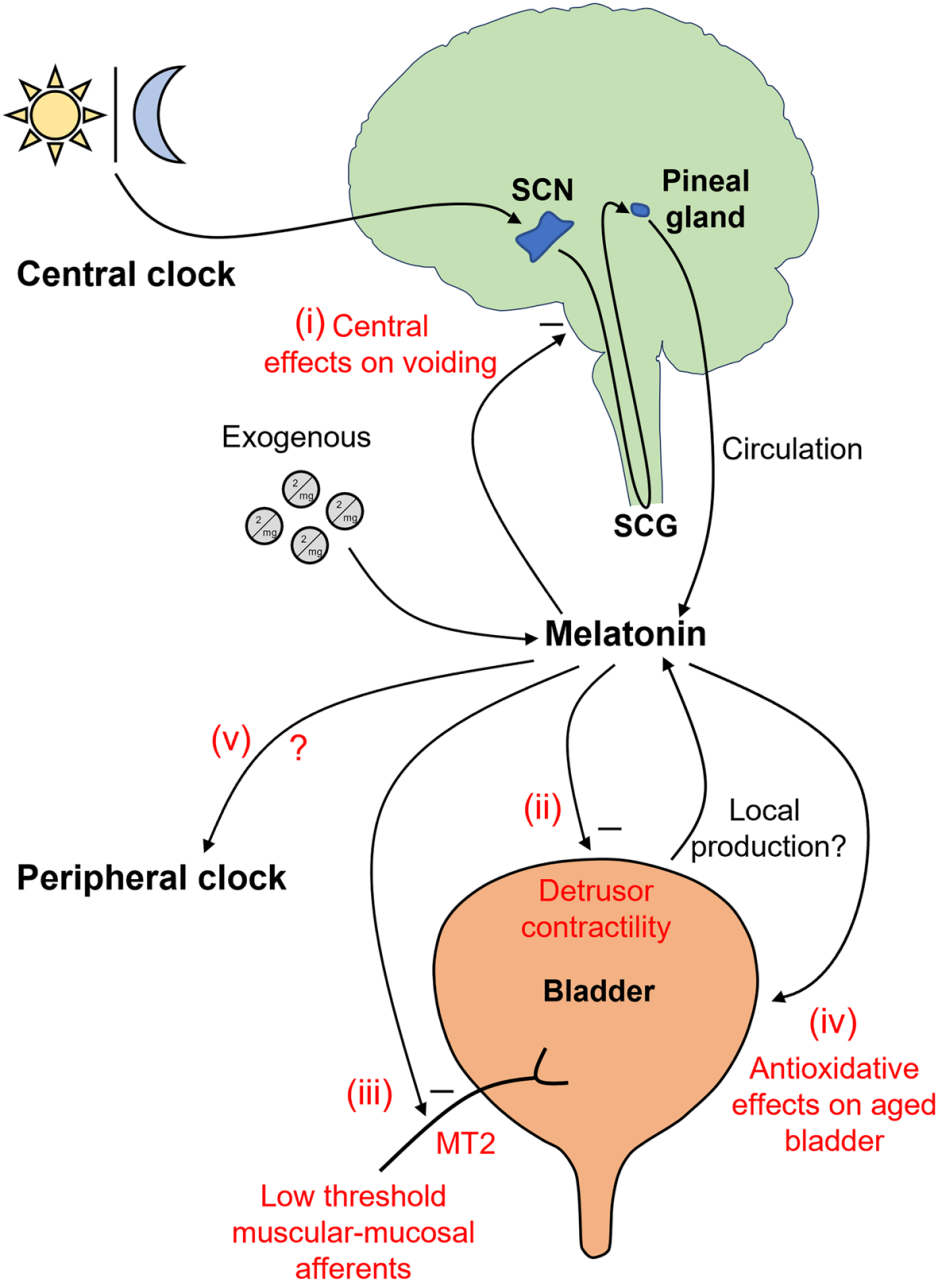
Proposed multiple mechanisms of melatonin effects on the bladder function. Melatonin is primarily produced by the pineal gland in response to the light dark cycle via the suprachiasmatic nucleus (SCN) and superior cervical ganglion (SCG) activation. Endogenously released melatonin from the pineal gland can centrally inhibit micturition pathways [(i): central effects on voiding via GABAergic system^19^]. Melatonin via circulation inhibits contractions and tone of the detrusor muscle [(ii): acting intracellularly but not via MT1/MT2 receptors^20^]. In addition, exogenously taken melatonin and possibly locally produced melatonin within the bladder (although, it is still unclear whether melatonin could be produced in the bladder apart from resident immune cells) can inhibit the mechanosensitivity of the low threshold muscular-mucosal afferents via G-protein coupled MT2 receptors [(iii): present findings] and ameliorate aged-related changes in bladder contractility [(iv): due to its antioxidative properties^60^]. It is possible that melatonin may also regulate clock genes in the bladder [(v): effect on peripheral clock, however, no data is available to date in the bladder].

The effect of melatonin on the bladder function is complex and may involve a variety of different mechanisms (Fig. 5). Melatonin, as master hormonal regulator of the circadian rhythms, may influence bladder function via central mechanism effects acting on the inhibitory GABAergic neurons in the brain. This action is likely of physiological significance since doses of centrally administered melatonin that affect bladder capacity are within the rage of melatonin fluctuation in the plasma^21,43^. In addition, melatonin has peripheral effects on the bladder contractility^22,33^, however, this requires higher doses than those found in the serum, demonstrating role of melatonin as promising pharmacological agent in modifying bladder contractility. As it was revealed in the present study, the peripheral effect of melatonin on bladder afferents is also an example of melatonin acting as pharmacological agent. These peripheral effects of melatonin are of great interest since it may be useful for treatment of common bladder disorders.

Nocturia (waking up at night more than once to void^55^), is a chronic condition affecting up to 75–90% of the elderly population^56^. The circadian rhythms disturbances including impaired circadian peripheral clock in the bladder, melatonin production, and sleep disturbance are suggested as main causes of the disease^4,6,20,51^. Endogenous melatonin levels decrease with age^57^. Furthermore, night serum melatonin level in elderly persons with nocturia is lower than those without^20^. Importantly, melatonin treatment significantly decreased nocturnal urination frequency and improved quality of life^20,53^. In the aged bladder, accumulation of reactive oxidative species is significantly augmented^58^ as well as stretch-induced firing of low threshold bladder afferents^59^, which may be responsible for development of lower urinary tract symptoms in the elderly. Interestingly, daily pre-treatment of the guinea pigs with melatonin restored age-related changes in bladder contractility in aged, but not adult, bladders through its effects on cytosolic and mitochondrial oxidative stress^60^. Taken together, we suggest the that ameliorating effect of melatonin on nocturia in the elderly could be due to multiple mechanisms: (i) its central effects of improving quality of sleep^52^ and bladder capacity^21^, (ii) inhibitory effects on detrusor muscle contractility (directly via intracellular mechanisms)^19,22^, (iii) inhibitory effects on bladder afferents (via MT2 receptors expressed on low threshold stretch-sensitive muscular-mucosal bladder afferents), and (iv) preventing age-related changes in bladder contractility due to its powerful antioxidative effects^60^ (Fig. 5). It is also possible that melatonin, acting as circadian rhythm synchroniser, may regulate directly peripheral clock genes in the bladder (v) (Fig. 5), as this was suggested in other tissues^61^. No data of possible effect of melatonin on peripheral clock genes in the bladder is available to date. More studies on aged animals are required to fully validate these proposed multiple mechanisms of action of melatonin in ameliorating nocturia in elderly.

In conclusion, melatonin directly inhibits the mechanosensitivity of low threshold stretch-sensitive muscular-mucosal bladder afferents acting via MT2, but not MT1, G-protein coupled receptors. This effect of melatonin is not due to its inhibitory action on detrusor muscle and may involve a decrease in cAMP levels in sensory nerve fibres. Melatonin has been proven to be beneficial in the treatment of many circadian related disorders including nocturia. Further research into the effects melatonin on mechano- and chemosensitivity of other classes of bladder afferents in normal and aged bladder are warranted.

## Methods

### Ethical approval

This study was approved by the Animal Welfare Committee of Flinders University (AEM1574-5) and performed in accordance with the Australian code for the care and use of animals for scientific purposes, 8th edition 2013 and the ARRIVE guidelines.

### Animals

Adult female guinea pigs (N = 44, weight 300–400 g) were housed in a 12 h:12 h light dark cycle with ad libitum access to a standard diet and water. The guinea pigs were euthanised by isoflurane inhalation overdose and severing of the spinal cord at the cervical level. This was performed between 0700–0730 hours to minimise circadian influence.

### Ex vivo bladder afferent preparation

This ex vivo bladder afferent preparation has been described in detail previously^26^. Briefly, the bladder and associated connective tissue containing the nerves was removed and opened into a flat sheet along the anterior bladder wall and placed in a modified Krebs solution consisting of (in mM): NaCl 118; KCl 4.74; NaH_2_PO_4_ 1.0; NaHCO_3_ 25; MgCl_2_ 1.2; CaCl_2_ 2.5; glucose 11 and nicardipine (3 µM) bubbled with 95% oxygen in 5% carbon dioxide. A rectangular full thickness sheet (approximately 12 mm by 15 mm) was formed and pinned mucosa up along the edge containing the nerves in a 22 ml organ bath containing warmed Krebs. The opposite side of the bladder was attached to a 12 mm custom-made stainless steel “rake”, also attached to a cantilever system with isotonic transducer (Harvard Bioscience 52-9511, S Natick, MA, USA). Counterweights (1–40 g) could be applied to the cantilever to distend the bladder while measuring the resulting changes in length. Fine nerve trunks entering the trigone were freed from the associated connective tissue, pinned, and placed in a paraffin oil bubble for electrical isolation. Each nerve trunk was then individually placed on a platinum electrode for recording. Electrical signals were amplified (DAM 80, WPI, USA), filtered via band-pass filter (BPF-932, CWE, USA, band pass 10 Hz–10 kHz) and recorded at 20 kHz using a data acquisition system (Micro 1401-4 CED, UK). Single units were discriminated offline by using Spike 2 software (version 10, CED, UK).

### Bladder afferent classes

Two major classes of bladder afferents were studied, high-responding mucosal, and low-threshold muscular-mucosal^34,35^. High-responding mucosal bladder afferents respond to mucosal stroking with von Frey hairs (10–500 mg), but do not respond to stretch of the bladder wall. These afferents are typically capsaicin-sensitive and have a long action potential duration. Low threshold muscular-mucosal bladder afferents respond to both mucosal stroking (10–500 mg) and bladder wall stretch (1–40 g) and typically have a short action potential duration^34,35^. Bladder afferent responses to stroking and/or stretch were used to determine the specific class of bladder afferents.

To facilitate drug penetration for either class of bladder afferent, a small hole (~ 2 × 2 mm) was formed adjacent to their receptive field using spring scissors. The mechanosensitivity of bladder afferents to mucosal stroking was determined using calibrated von Frey hairs (10, 100 and 500 mg) stroked across a hot spot area at a rate of 5 mm s^−1^ 5 times with 30 s intervals. The mechanosensitivity of bladder afferents to stretch was determined by adding weights (1–40 g) to the cantilever for a period of ten seconds followed by a one-minute rest period before a heavier weight was added.

### Effect of melatonin and melatonin receptor antagonists on the mechanosensitivity of bladder afferents

After initial recordings, melatonin was added to the Krebs organ bath over the receptive field of the afferent and equilibrated for 5 min. The mechanosensitivity of the bladder afferents was then redetermined. This was repeated for increasing concentrations of melatonin (0.3, 1, 3, 10, 30 and 100 µM). Comparable time-control experiments were performed in the absence of melatonin which did not show and significant effects.

In separate experiments melatonin was combined with antagonists of MT1 (S26131 10 µM for 30 min) or MT2 (10 µM K-185 or 4-P-PDOT for 30 min). The initial effect of melatonin (30 µM) on the mechanosensitivity of bladder afferents was determined, washed out, followed by application of the antagonist alone, and then the effect of melatonin combined with the antagonist.

### Effect of melatonin and forskolin on the mechanosensitivity of bladder afferents

After initial baseline measurements, forskolin was added to the organ bath and incubated for a period of 30 min before re-establishing the mechanosensitivity of bladder afferents. The effects of forskolin did not wash out. Therefore, to determine the effects of melatonin on the forskolin action, in a separate set of experiments, melatonin was added to the organ bath and equilibrated for 10 min, after which the mechanosensitivity of the bladder afferents was determined. Following this, forskolin (10 µM) was added to the organ bath and equilibrated for 30 min. Measurements of mechanosensitivity were taken every 10 min during this incubation to determine whether melatonin can prevent the effects of forskolin on bladder afferents.

### Drugs

Stock solutions (10 mM) of melatonin were made using PBS and stock solutions of S26131, K-185, 4-P-PDOT, and forskolin were made using DMSO. All drugs were stored at − 20 °C and diluted to final concentration immediately before experiments. Preliminary DMSO (0.1%) control studies were carried out and no significant effects were found on the mechanosensitivity of the studied bladder afferents. Melatonin (M250), K-185 (K1888), 4-P-PDOT (SML1189), and forskolin (F6886) were obtained from Sigma-Aldrich Australia, and S26131 (HY-122136) was obtained from MedChemExpress USA.

### Data analysis

All data is presented as the mean ± SEM, with n referring to the number of afferents and N to number of animals. Analysis was performed using GraphPad Prism 9 software. All data was analyzed using a one-way ANOVA with Tukey’s post hoc test. P-values were less than 0.05 were considered significant.

## Data Availability

The data sets analysed during the current study are available from the corresponding author on reasonable request.
